# Single-centre experience of laparoscopic nephrectomy: Impact of learning curve on outcome

**DOI:** 10.4103/0970-1591.33719

**Published:** 2007

**Authors:** Mrigank S. Jha, Nitin Gupta, Saurabh Agrawal, M. S. Ansari, Deepak Dubey, Anil Mandhani, Aneesh Srivastava, Anant Kumar, Rakesh Kapoor

**Affiliations:** Department of Urology and Renal Transplantation, Sanjay Gandhi Post Graduate Institute of Medical Sciences, Lucknow - 226 014, UP, India

**Keywords:** Laparoscopic, learning curve, nephrectomy

## Abstract

**Aim:**

To present our experience of laparoscopic nephrectomies done for benign and malignant conditions; and the impact of learning curve on outcome.

**Settings and Design:**

Retrospective study.

**Materials and Methods:**

Between January 2000 and September 2006, 396 laparoscopic nephrectomies were performed at our institute for various benign and malignant conditions. These included 250 simple nephrectomies, 48 nephroureterectomies, 95 radical nephrectomies, two partial nephrectomies and one hemi-nephrectomy. For the purpose of self-evaluation, we have divided our experience into two groups. Group 1 (learning phase) comprised the first 100 cases; Group 2 (consolidation phase) comprised cases performed after the initial learning phase. Retrospective evaluation of the case records was done to evaluate the differences in the operative and postoperative outcome.

**Statistical Analysis Used:**

Student's ‘t’ test using SPSS 14.0 software.

**Results:**

Demographic profile of the patients and relative indications of procedures performed were similar in the two groups. Mean operative time in Group 1 was 262 ± 37 min, which reduced to 184 ± 44 min in Group 2 (*P*<0.001). Mean operative blood loss was 310 ± 58 ml and 198 ± 88 ml (*P*<0.001); and blood transfusion was required in 38% and 13.5% of patients (*P*<0.001) of Group 1 and Group 2 respectively. There was a significant reduction in the intraoperative and postoperative complications from 16% in Group 1 to 3.4% in Group 2 (*P*<0.001). Similarly, conversion to an open procedure was required in 17% cases of Group 1 and 5.4% cases of Group 2 (*P*<0.01).

**Conclusions:**

Laparoscopic nephrectomy is a viable option which can be performed safely with increasing experience.

Laparoscopic nephrectomy for benign renal disease was initially described in 1991 by Clayman *et al.*[1] Advantages of the laparoscopic approach are decreased morbidity, shorter hospital stay, rapid convalescence and better cosmesis. Encouraged by these advantages of laparoscopic procedures, many urological centers across the world are routinely performing more extensive and complicated procedures via this approach.

Laparoscopic nephrectomy was initiated at our institution in January 2000. We reviewed our series of laparoscopic nephrectomies done for various benign and malignant conditions and tried to find the impact of the learning curve on the outcome.

## MATERIALS AND METHODS

Between January 2000 and September 2006, 396 laparoscopic nephrectomies were performed at our institute. These included 250 simple nephrectomies (LSN) for nonfunctioning kidney, 48 nephroureterectomies (LNU) for vesicoureteric reflux with nonfunctioning kidney or transitional cell carcinoma, 95 radical nephrectomies (LRN) for renal cell carcinoma, two partial nephrectomies (LPN) for small (< 4.0 cm) exophytic renal tumors and one heminephrectomy for a duplex system with a nonfunctioning moiety. The approach was transperitoneal in the majority of cases according to the technique described by Clayman *et al.*,[1] however, 37 nephrectomies were performed by the retroperitoneal approach as described by Gaur,[2] with minor variations in both the procedures depending upon the preference of the operating surgeon.

We retrospectively reviewed the case records of these patients to obtain the relevant intraoperative and postoperative data which included operative time, blood loss, need for blood transfusion, conversion to open procedure, significant intraoperative and postoperative complications, hospital stay and overall cost.

For the purpose of self-evaluation, we divided our experience into two groups. Group 1 (learning phase) comprised the first 100 cases and Group 2 (consolidation phase) comprised cases performed after the initial learning phase.

The results were analyzed using Student's ‘t’ test for continuous variables. SPSS version 14.0 was used for statistical analysis.

## RESULTS

In the learning phase (Group 1), 59 LSN, 11 LNU and 30 LRN were done; while in the consolidation phase (Group 2), the cases done included 191 LSN, 37 LNU, 65 LRN, two LPN and one heminephrectomy. The demographic profile of the patients in these two groups was comparable [Table 1]. The indications for the surgeries performed were as given in Table 2.

**Table 1:** Demographic profile of patients in the two groups

Characteristics	Group 1 (n=100)	Group 2 (n=296)	*P* value
Sex (male: female)	68:32	198:98	ns
Age range in years (mean)	12–68(32)	9–72(35)	ns
Side (left: right)	56:44	182:114	ns
Approach (Transperitoneal:Retroperitoneal)	91:9	268:28	ns

**Table 2:** Indications for nephrectomy

Diagnosis	Group 1 (n=100)	Group 2 (n=296)
Nonfunctioning kidney	59	191
VUR with nonfunctioning kidney	10	25
Renal cell carcinoma	30	67
TCC upper tract	1	13

VUR: Vesicoureteric reflux

The mean operative time for Group 1 was 262±37 (210-320) min, which was significantly longer than the mean operative time for Group 2 of 184±44 (120-280) min. Overall, patients undergoing laparoscopic procedures in Group 1 had significantly higher mean blood loss (310±58 ml vs. 198±88 ml) and had higher blood transfusion requirement (38% vs. 13.5%). The mean hospital stay in the two groups was however similar (5.2 days vs. 4.8 days) [Table 3].

**Table 3:** Clinical outcome of patients in the two groups

Variable	Group 1 (n=100)	Group 2 (n=296)	*P* value
Mean operative time (minutes)	262±37	184±44	<0.001
Mean blood loss (ml)	310±58	198±88	<0.001
Blood transfusion (%)	38 (38%)	40 (13.5%)	<0.001
Mean hospital stay (days)	5.2±0.4	4.8±0.5	ns
Conversion to open (%)	17 (17%)	16 (5.4%)	<0.001

A learning curve effect of laparoscopic nephrectomy was clearly evident in all the clinical parameters including the complication rate. Significant number of complications (16) occurred in Group 1, wherein six cases had severe bleeding from the renal hilum which could not be controlled laparoscopically and conversion to open was required. Of these, one patient had adrenal vein tear and another one had avulsion of lumbar vein, both of which were ligated. Four patients had bleeding from the renal vein stump which was repaired after clamping. In addition to this, two cases had mesocolic vessel injury and one had intestinal injury which also made conversion to open surgery necessary. After opening, mesocolic vessels were ligated and defect closed and similarly the intestinal injury (ileum) was repaired. Other minor complications encountered in Group 1 included splenic tear in one patient which was managed by surgicel application, serosal tear in one and kidney fracture in two which were all managed laparoscopically. In the postoperative period, two patients had wound infection at the site of kidney retrieval and another one developed port site hernia at follow-up [Table 4].

**Table 4:** Complications in the two groups

Complication	Group 1 (n=100)	Group 2 (n=296)
Major		
Death	0	1
Renal vessel bleeding	6	5
Intestinal injury	1	0
Mesenteric vessel injury	2	0
Diaphragmatic injury	0	2
Minor		
Splenic tear	1	0
Serosal tear	1	1
Wound infection	2	0
Kidney fracture	2	1
Port site hernia	1	0
Total (%)	16 (16%)*	10 (3.4%)*

* *P* < 0.001

**Table 5:** Reasons for conversion to an open procedure

Reason for conversion	Group 1 (n=100)	Group 2 (n=296)
Renal vessel bleeding	6	4
Intestinal injury	1	0
Mesenteric injury	2	0
Diaphragmatic injury	0	1
Inability to proceed		
Instrument malfunction	3	1
Adhesions	5	10
Total (%)	17 (17%)	16 (5.4%)

As opposed to the high complication rate in Group 1, only 3.4% of the cases performed in Group 2 had significant intraoperative or postoperative complications. The major complications encountered included renal vein stump bleeding in five cases of which four required conversion to open surgery to control bleeding while in one it was managed laparoscopically. In this phase, renal vein was mainly controlled by hemolock, with staples used in two cases. Diaphragmatic injury occurred in two cases— the first was a case of simple nephrectomy during which there was a small rent in the diaphragm which was sutured laparoscopically. The other patient was a case of RCC with infiltration of diaphragm which necessitated conversion to open surgery and repair of diaphragm. Minor complications encountered in this group were serosal tear and kidney fracture in one patient each [Table 4].

Other than the major complications, conversion was required as a result of inability to proceed due to severe adhesions in five cases in Group 1 and 10 in Group 2. Instrument malfunction was responsible for conversion to open surgery in three cases in Group 1 and one case in Group 2 and included significant gas leak due to trocar problems; camera and monitor problems; and stapler malfunction [Table 5].

There were no intraoperative deaths. One patient died in the postoperative period in Group 2. He was a 58-year-old male with nonfunctioning kidney with no comorbidities for which LSN was done. The surgery was uneventful; however, in the immediate postoperative period patient had a massive myocardial infarction and expired.

The overall expenditure of patients undergoing laparoscopic nephrectomy at our institute ranged from Rs. 22,000 to 48,000 (mean Rs. 28,000± 3000).

## DISCUSSION

The first reported use of laparoscopy in urology in 1991[1] by Clayman for nephrectomy fueled interest in performing renal surgery via this route and broadened the indication of laparoscopic surgery to virtually all genitourinary pathologies. Since then laparoscopic nephrectomy has gained momentum as an adequate mode of treatment in appropriately selected patients. Even in the early years of the learning curve, it was consistently revealed that laparoscopic nephrectomy is as effective as open surgical extirpation and is better tolerated than open surgery.[3,4] The advantages of laparoscopic nephrectomy are less postoperative pain with decreased need for analgesics and a shorter convalescence period. In addition, since the incisions required for laparoscopic procedures are markedly smaller than a regular flank incision, the risk of wound weakness and herniation is lessened[5,6] and it gives better cosmetic results. It is currently being performed either by the transperitoneal or retroperitoneal approach.

While there are obvious advantages of laparoscopic surgery, there are also unique challenges and complications associated with laparoscopy for renal surgery. So it is important that urologists should continue to learn from the past laparoscopic complications to avoid repeating the same mistakes when possible. Laparoscopic nephrectomy is being performed at our institute for more than six years and nearly 400 nephrectomies have been done successfully. This provides a good database to review the clinical outcomes and complications encountered, especially the trend over time.

Laparoscopic skills evolve with repetition and a slow learning curve exists for achieving these skills. In a recent review of laparoscopic urological complications, it was suggested that a minimum of 50 difficult cases are required to achieve adequate laparoscopic skills and complications decrease with increasing experience.[7] This is truly evident in two recent multi-institutional reviews of laparoscopic urological surgeries.[7,8] In the first multicentric study, in the first 100 laparoscopic cases that were completed at each institution, the complication rate was 13.3%, which subsequently decreased to 3.6% for the remaining cases performed later. In the second study, similar results were reported with a complication rate of 9% for the first 100 cases and 4% for the next 250 cases.[8] In our series, the complication rate was 16% in the first 100 cases and this rate was subsequently reduced to 3.4% in the next 296 cases.

An important point to be considered here is that laparoscopic nephrectomy at our institute is being done by six different surgeons, each with a different level of experience and expertise. The results presented, therefore, indicate the cumulative outcome of all the surgeons. This could probably also explain the high complication rate in the first phase when the new surgeons were just joining in. However, with increasing experience the complication rate reduced for all of them as is easily seen by the difference in the two phases. Additionally, in the initial phase only metallic clips were used to gain control of the renal vasculature; while in later stages, hemolocks and occasionally vascular staplers were being used which have helped us in bringing down the rate of vascular events. Overall, the complication rates in our series also compare well with another large multi-institutional study where out of 185 patients undergoing laparoscopic nephrectomy, 16% had complications and 5% required open conversion.[9]

The impact of experience is especially evident if the rates of need to conversion to open procedure are compared in the two groups (17% vs. 5.4%). Emergency open conversion was most commonly done in cases of uncontrollable hemorrhage, the other important reason being irreparable injury to the diaphragm or surrounding viscera. The decision to resort to open conversion due to bleeding should be made within seconds because even the opening process until temporary control of the bleeding is achieved may still require an additional two to five minutes. Elective conversion was the result of lack of progression due to instrument malfunction or extensive adhesions, especially in cases of simple nephrectomy, these procedures being difficult due to scarring, inflammation and loss of anatomical planes.

Laparoscopic nephrectomy often takes more operative time. However, the impact of experience underlies the importance of the learning curve to achieve acceptable time. In a study in 1996,[4] the initial operative time for laparoscopic nephrectomy was 6.9h which decreased to 5.5h in the later series. Similar results were reported in yet another study conducted in 1998.[10] In the present study, likewise, operative time decreased by nearly 80min when comparing the two groups (initial 262 min to 184 min later).

Economically, in developing countries laparoscopic procedures are considered to be costlier than the open procedures. The increased cost may be attributable to initial longer operating time and use of expensive instruments. However, the advantages in terms of short convalescence, better cosmesis and minimal scar clearly outweigh the difference in cost[11] making it feasible even in poorer nations.

With our experience in performing laparoscopic nephrectomies in nearly 400 cases, we would like to like to propose certain guidelines for selecting cases in the initial part of the learning curve:

1. One must be well read regarding the theoretical, anatomical and physiological aspects of laparoscopic abdominal surgery.
2. Observe and assist in at least 20 cases regarding port placement and instrument handling.
3. Start with reflection of the colon and later mobilization of the kidney after control of the renal vessels in the presence of an expert.
4. Laparoscopic nephrectomies on the left side are easier to perform in the initial part of the learning curve.
5. Laparoscopic radical nephrectomy for small (<7 cm) renal tumors away from the hilum is easier to perform than a simple nephrectomy for an infected nonfunctioning kidney.
6. Conversion to open surgery should be in the best interest of the patient. It is a learning experience and never a failure of laparoscopy.

## CONCLUSIONS

Laparoscopic nephrectomy is a well-established and technically feasible modality for the treatment of both benign and malignant renal conditions and can be mastered with increasing experience.
